# Is it time to scrap Scadding and adopt computed tomography for initial evaluation of sarcoidosis?

**DOI:** 10.12688/f1000research.11068.1

**Published:** 2018-05-16

**Authors:** Andrew Levy, Nabeel Hamzeh, Lisa A. Maier

**Affiliations:** 1 Division of Environmental and Occupational Health Sciences, Department of Medicine, National Jewish Health, Denver, CO, USA; 2 Division of Pulmonary and Critical Care Sciences, Department of Medicine, University of Colorado School of Medicine, Denver, CO, USA; 3 Environmental and Occupational Health Department, Colorado School of Public Health, University of Colorado, Denver, CO, USA

**Keywords:** sarcoidosis, high-resolution computed tomography, chest X-ray, diagnosis

## Abstract

In this review, we argue for the use of high-resolution computed tomography (HRCT) over chest X-ray in the initial evaluation of patients with sarcoidosis. Chest X-ray, which has long been used to classify disease severity and offer prognostication in sarcoidosis, has clear limitations compared with HRCT, including wider interobserver variability, a looser association with lung function, and poorer sensitivity to detect important lung manifestations of sarcoidosis. In addition, HRCT offers a diagnostic advantage, as it better depicts targets for biopsy, such as mediastinal/hilar lymphadenopathy and focal parenchymal disease. Newer data suggest that specific HRCT findings may be associated with important prognostic outcomes, such as increased mortality. As we elaborate in this update, we strongly recommend the use of HRCT in the initial evaluation of the patient with sarcoidosis.

## Background

Sarcoidosis is a systemic granulomatous disease of unclear etiology that affects the lungs and thoracic lymph nodes in over 90% of patients. The disease’s onset, course, and organ involvement are highly variable. Presentations can range from asymptomatic disease incidentally noted on chest imaging and acute disease with total resolution to chronic progressive disease resulting in end organ damage, such as pulmonary fibrosis, and death. In fact, chronic pulmonary disease and fibrosis are major causes of death in patients with sarcoidosis ^1^. The diagnosis is generally based on clinical and radiographic findings, granulomas on biopsy, and exclusion of other granulomatous diseases, such as fungal disease, tuberculosis, and chronic beryllium disease. Classically, the chest radiograph has been used for the diagnosis and prognostication of sarcoidosis in patients with suspected disease ^2^. Below, we compare the limitations and advantages of chest radiography versus computed tomography (CT) for the initial evaluation of pulmonary sarcoidosis.

Bilateral hilar lymphadenopathy is the most common finding on chest X-ray (CXR). Lung parenchymal findings are myriad, ranging from nodules and consolidations to irreversible fibrosis ^3^. Scadding classified thoracic disease on CXR into five stages as described in Table 1 ^4^. Staging was derived for prognostic purposes and does not represent a natural progression of pulmonary disease in sarcoidosis. Scadding reported that as the chest radiographic stage increased, there was a lower likelihood of spontaneous remission without treatment. Over five decades later, this staging system is still used to classify patients with sarcoidosis clinically, for research studies and to provide prognostic information to patients, in part because of its simplicity ^4– 6^. The ubiquity, cheap cost, and low radiation of chest radiography have also contributed to the wide use of Scadding staging.

**Table 1.** Scadding staging for pulmonary sarcoidosis.

Stage	Chest radiograph findings
0	No chest abnormality
I	Hilar lymphadenopathy
II	Hilar lymphadenopathy and parenchymal abnormality
III	Parenchymal abnormality without hilar lymphadenopathy
IV	Fibrosis with volume loss

There have been no recent recommendations to help guide the use of chest imaging; as of 1999, the official American Thoracic Society guidelines recommended CXR for initial evaluation and to preserve CT scan for patients with atypical clinical or X-ray findings or for suspicion of a complication of pulmonary disease, such as bronchiectasis or fungal superinfection ^7^. It is not clear whether these recommendations are still appropriate or still being followed as patients receive CT scans for many routine clinical evaluations, in the emergency room and the doctor’s office.

## Limitations of the chest radiograph

Despite past recommendations and current wide application, the use of CXR for initial evaluation and prognostication of pulmonary sarcoidosis has clear limitations. Scadding himself recognized that his staging was useful to predict prognosis in stage I and IV disease but that there was no significant difference in prognosis between stage II and III ^4^. We have noted this in our clinic as well and this is due in part to the following limitations.

### Impaired interobserver variability

There is significant variation in the interpretation of X-ray, even among chest radiologists. Using data from a clinical trial, Baughman *et al*. analyzed the interobserver variability between two chest radiologists comparing Scadding CXR staging at initial evaluation ^8^. The authors found only fair interobserver concordance overall (weighted κ = 0.43) and fair interobserver concordance with regard to the presence of fibrosis (weighted κ = 0.51). They also noted that their chest radiologists had difficulty in agreeing on the presence of hilar lymphadenopathy and thus distinguishing stage II and III disease ^8^.

### Poor predictor of lung function

Chest radiography has proven to be an unreliable predictor of derangements in lung function on initial evaluation. Frequency of airflow limitation generally increases with increasing stage; however, there is significant overlap between stages and, again, no significant difference between stage II and III disease ^9^. CXR staging is especially poor in predicting exercise-induced desaturation and thus oxygen levels and, in one study, offered no further prognostic value when combined with pulmonary function test parameters, such as the diffusing capacity of the lung for carbon monoxide (DLCO) ^10^.

### Missed parenchymal disease

**Figure 1.**
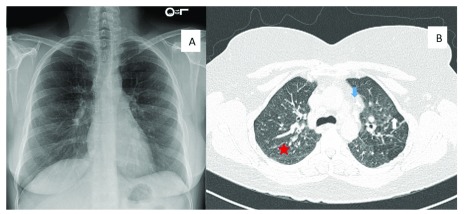
A chest radiograph and high-resolution computed tomography from the same patient are depicted. ( **A**) The chest radiograph would be classified as stage III, as there is mild parenchymal abnormalities but no lymphadenopathy. ( **B**) The high-resolution computed tomography demonstrates numerous nodules that track along the bronchovascular bundle bilaterally (red star), lymphadenopathy (blue arrow), and areas of ground glass opacity (white arrow).

**Figure 2.**
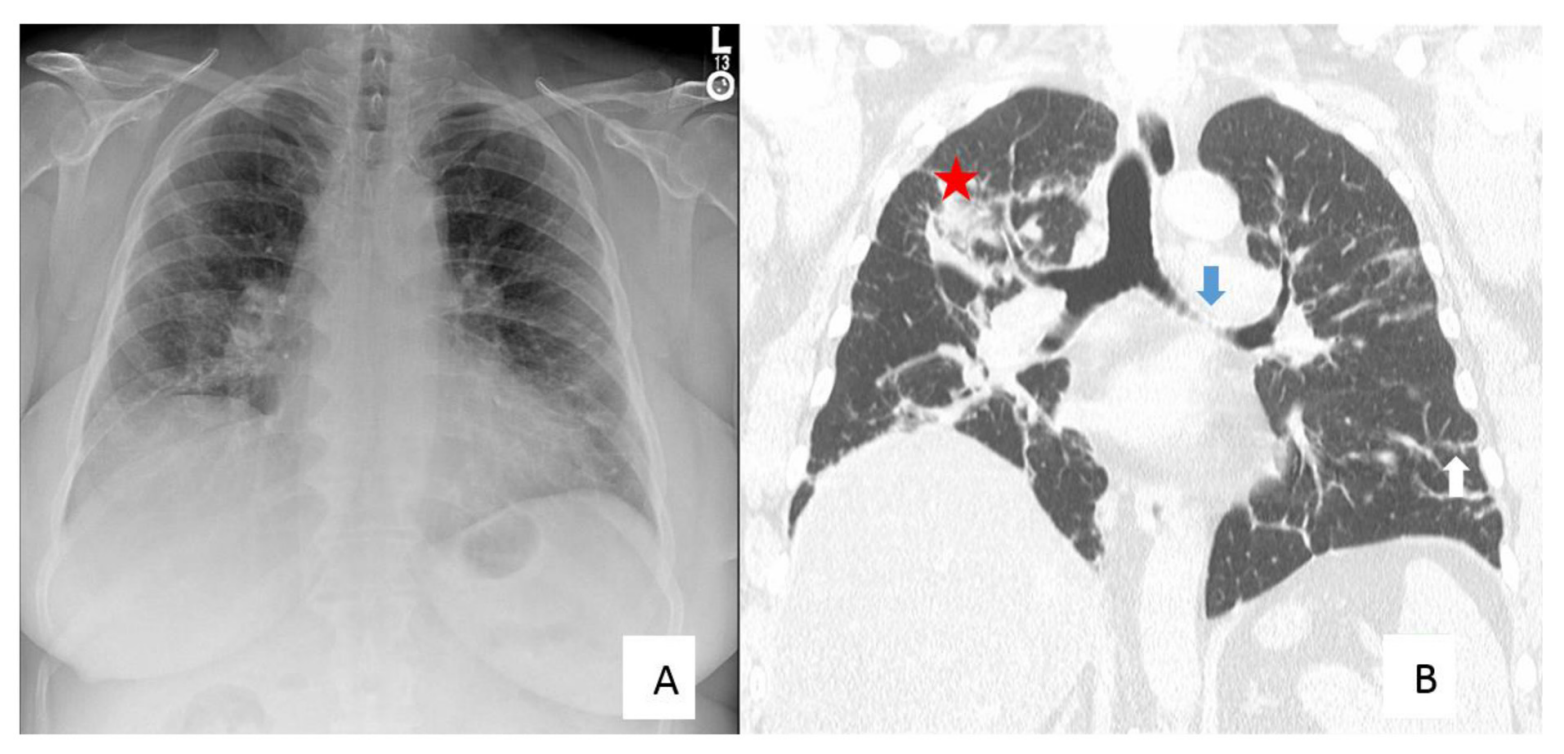
A chest X-ray and high-resolution computed tomography from the same patient are depicted. ( **A**) The chest X-ray was interpreted as Scadding stage III. ( **B**) Computed tomography clearly demonstrates findings consistent with fibrosis, including bronchovascular distortion (blue arrow), reticulation (white arrow), and conglomerate masses (red star). These are findings that are seen in more advanced fibrotic sarcoidosis.

Chest radiography has a lower sensitivity for lung parenchymal disease compared with high-resolution computed tomography (HRCT) of the chest. Multiple studies have demonstrated significant rates of parenchymal disease on HRCT in patients with either Scadding stage 0 (no disease) or Scadding stage I (nodal disease only) on initial X-ray evaluation ^11, 12^. Often, patients with stage 0 or I disease have demonstrated pulmonary impairment on lung function testing, suggesting that these patients have parenchymal or airway disease that has not been captured by CXR ^9^. Indeed, in our clinical experience, we have noted limited prognostic value for CXR as well as poor depiction of important parenchymal findings that are visible on corresponding HRCT, as illustrated in Figure 1. We have also noted fibrotic findings on CT that were not seen on CXR, as seen in the radiograph in Figure 2.

## Advantages of high-resolution computed tomography

Based on our review and interpretation of the literature, we posit that HRCT may be a better method for the initial evaluation of patients with suspected or confirmed sarcoidosis. HRCT is able to evaluate the lungs and airways with much higher resolution than CXR, in multiple planes, and offers clinicians improved sensitivity and specificity for detecting lung abnormalities ^2^.

Classically, HRCT reveals bilateral hilar lymphadenopathy and micronodules in the lung parenchyma with a perilymphatic distribution ( Figure 1). Lung findings are often more prominent in the upper lung fields. HRCT can also detect specific patterns and distribution of parenchymal and airway abnormalities that can have clinical implications. Bronchial thickening or narrowing can be detected by HRCT and is usually predictive of granulomatous disease of the airway seen on fiberoptic survey. Extrinsic findings, such as traction bronchiectasis and bronchovascular distortion, can be detected on CT. Mosaic attenuation seen on CT can be predictive of air trapping, an indicator of airway disease ^13^.

HRCT is more sensitive in revealing fibrosis and diagnosing end-stage disease. In various studies, fibrosis has been seen in 20–50% of patients, which is much higher than the 5–10% rate estimated by using CXR on initial evaluation ^14^. Detection of an increased frequency of fibrosis in these patients does not appear to be a trivial finding, as the presence and the pattern of fibrosis are associated with more progressive derangements in lung function, as discussed below. In addition to having a higher sensitivity of lung parenchymal findings, HRCT is better able to demonstrate lymphadenopathy and findings consistent with pulmonary hypertension, a potential complication of sarcoidosis.

### Improved interobserver reliability

In one study of 80 patients with sarcoidosis, there was variable interobserver reliability (κ = 0.36–0.78) between chest radiologists for specific findings on HRCT, such as bronchovascular bundle thickening, reticulation, and consolidation ^11^. However, when HRCTs were evaluated using a validated semi-quantitative score of overall severity derived by Oberstein *et al*. ^12^, there was excellent interobserver reliability (κ = 0.99).

### Association with lung function

The use of a standardized assessment of HRCT (Oberstein score) not only proved reproducible but also demonstrated a statistically significant correlation with derangements in functional parameters, such as forced expiratory volume in 1 second (FEV _1_), forced vital capacity (FVC), and DLCO as well as resting and exertional arterial oxygenation ^11^. Not only is overall severity associated with decreased lung function but also specific findings on HRCT are predictive of different patterns of abnormal physiology. Abehsera *et al*. described the HRCT findings in a cohort of patients with end-stage disease and identified three distinct patterns of fibrosis in these patients: architectural distortion, linear fibrosis, and honeycombing ^15^. Architectural distortion was associated with obstruction on pulmonary function tests, whereas honeycombing was associated with restriction and reduction in DLCO. Linear fibrosis was not associated with functional impairment ^15^; nothing similar has been noted with Scadding stage alone.

### Diagnostic and prognostic value

Sarcoidosis is increasingly diagnosed via endobronchial ultrasound (EBUS)-guided biopsy, which has increased the value of HRCT and its ability to identify the exact size and location of mediastinal and hilar lymph nodes ^16, 17^. This often spares patients the morbidity of surgical biopsy. Detecting patterns of lung disease on HRCT also allows better evaluation of active versus irreversible lung disease. Oberstein *et al*. were able to detect higher inflammatory markers in both serum and bronchoalveolar lavage associated with intrapulmonary nodules and involvement of the bronchovascular bundle ^12^. This may guide treatment decisions in the future, although further investigation is necessary. As noted above, HRCT has higher sensitivity in detecting fibrotic disease as compared with CXR and this may have implications for treatment or treatment response or both.

### Findings on high-resolution computed tomography may also have implications for patient mortality

In a retrospective study, Walsh *et al*. derived an algorithm of independent variables that predicted mortality in patients with pulmonary sarcoidosis ^18^. They found that two CT variables—fibrosis in 20% of lung fields and pulmonary artery diameter—as well as a composite of physiologic variables produced a staging system to predict prognosis in patients with pulmonary sarcoidosis ^18^. Whether these results will be confirmed in other sarcoidosis groups or whether there are other uses of CT needs to be determined. Death is usually a distant occurrence in patients with sarcoidosis, so other outcomes, such as change in pulmonary function or exercise capacity, may further highlight the prognostic value of HRCT.

## Conclusions

Current guidelines for the initial evaluation of sarcoidosis are stuck in the 1960s, when Scadding staging with CXR was first derived, recommending CXR for initial evaluation of patients diagnosed with sarcoidosis. We recommend HRCT of the chest during initial evaluation, as it offers better definition of lung parenchymal and airway abnormalities as well as vascular structures (which were beyond the discussion we could undertake in this article), detection of reversible versus irreversible disease, and some prediction of disease course. We recognize the risks of HRCT, including radiation and the possibility of unnecessary workup for incidental findings. For follow-up, patients with remitting or stable disease would be well served by a yearly CXR, although when there are changes in clinical course or suspicion for new complications of their underlying disease, repeat HRCT may be of use. As we further understand abnormalities in pulmonary sarcoidosis and develop new targeted treatments, HRCT and its ability to depict specific lesions and patterns may gain even greater value. With the aid of further research, it is time to come up with a new, uniform, staging system that takes into account specific CT patterns and helps prognosticate disease course, pulmonary impairment, and treatment response. Having a baseline CT for patients now may become even more important for a patient’s treatment in the future and will move our current care of these patients into the 21st century.
